# Decolonising our minds: what UK psychiatry needs to (un)learn

**DOI:** 10.1192/bjb.2024.62

**Published:** 2024-08

**Authors:** Venkatalakshmi Satram

**Affiliations:** Imperial College London, London, UK

**Keywords:** Transcultural psychiatry, decolonising mental health, social, political and economic determinants of mental health, collective responsibility and social justice, contextualised care.

## Abstract

The dominance of Western orthodox models of psychiatry has led to colonisation of the mind and marginalisation of diverse cultural conceptualisations of distress and forms of caring. Compounded by a crisis of care and chronic underfunding, this has also diminished our practice here in the UK. This article probes the biases that UK psychiatry must unlearn and what lessons it can learn from decolonising praxes originating in the Global South. This is a call to action. We must transform our mindsets and advocate for contextualised, collective, integrative and socially just mental healthcare in the UK.

UK psychiatry, within the context of the National Health Service (NHS), is on the brink of collapse, amid the current crisis of care. The priorities of people in our community and the psychiatrists caring for them have been superseded by the priorities of politicians.^1^ Chronic lack of funding, alongside ideologies of Western orthodox psychiatry, have created a culture of moral injury^2^ among clinicians and policymakers, where many may be desensitised to the problems of the system.

There has been a drive to universalise Western psychiatric ideologies, through the Movement for Global Mental Health (MGMH).^3^ The colonial scale-up from the Global North into the Global South is strongly challenged by many communities around the world.^3^ The ways different cultures have been responding to Western psychiatry need to be brought back, in full circle, to the UK, where crucial discussions and applications of transcultural psychiatry need to take place.

So, what biases must UK psychiatry unlearn, to be open to the diverse ways of conceptualising and caring for people experiencing distress? How can we rehumanise and decolonise psychiatry, through lessons from the Global South?

## Challenging individualism: contextualising distress

Western orthodox psychiatry, amplified through prolonged lack of funding and time, has created a UK psychiatric system that is discriminatory, hyper-individualistic and reductive. The little available funding is rationed towards diagnosing those who are ‘sick enough’, ‘high risk’ enough and/or ‘medically cleared’.^2^ People are too often plucked out of their contexts and objectified as symptoms existing in a vacuum. When people are decontextualised from their entire lived experience, their symptoms of distress are depoliticised, pathologised and forced to fit into Eurocentric ‘mental illness’ diagnoses. This reductionism is also reflected on a larger scale. In a society dominated by neo-liberal ideology,^4^ medicine is viewed as a business, healthcare as a transaction, patients as customers^5^ and compassion as a commodity.

Radical reconceptualisations of diagnostic labels in the Global South, partially in response to the Eurocentric diagnostic manuals, such as the DSM, shed light on the shortcomings of the UK psychiatric system. In Japan, the label of schizophrenia, known as *Seishin-Bunretsu-Byo* (mind-split-disease), caused severe stigma, which worsened social isolation and consequently dehumanised patients, as they were seen as a lost cause.^6^ Clinicians were reluctant to diagnose patients, resulting in patients not being able to, or wanting to, access the care they needed.^6^ The term was reclaimed and reimagined as *Togo-Shitcho-Sho* (integration disorder) to change mindsets about schizophrenia, by depicting the condition as a syndrome.^6^ This reflects a dynamic understanding of the condition, responding to experiences of distress and stigma within the Japanese community. This renaming not only altered personal and public perceptions, but also transformed the clinician–patient relationship. The number of people informed of their diagnosis increased, allowing for greater optimism and education about the tailored care available. Social isolation from core familial relationships also reduced.^6^

Medicalising and pathologising life's distress, through focusing narrowly on the individual, fails to address the contributing socio-politico-economic injustices. This can ultimately cause more harm than good. The Ubuntu philosophy, the ‘African conception of humanism’, recognises the deep interdependencies of people, where personhood exists within a community.^7^

Adopting an Ubuntu-centred African feminist approach, the African Institute for Integrated Responses to Violence against Women and Girls and HIV/AIDS (AIR), in the Democratic Republic of Congo, applies these principles to the psychiatric diagnosis of post-traumatic stress disorder (PTSD) and reveals how narrow a category this is to truly describe trauma.^8^ For those born into discrimination and marginalisation, distress and trauma are a common constant throughout life, as ‘the world itself is the stressor’.^8^ Trauma is repoliticised and recognised as being caused by structural power relations. One's distress affects and is affected by others, thus shifting the notion of the individual self to the collective self. This highlights the bidirectionality of care, where immersion in the stories of those experiencing distress can inspire clinicians, suggesting ways they can also deal with stressors in their own contexts and build resilience. In this way, power dynamics in the clinician–patient relationship can be challenged, and patient expertise and indigenous wisdoms beyond Western orthodoxies can be valued.^8^

## Embracing the collective: possibilities of healing

Within the structure of UK psychiatry, time is not afforded to building deep authentic caring relationships between clinicians and patients, let alone within the larger community. The fragmented system of functional teams means there are multiple professionals and complex care pathways for patients to navigate. A patient may see three or four consultant psychiatrists within one period of acute illness, meaning that stable, long-lasting communities of care cannot be formed around the patient.^1^ The siloed nature of mental healthcare also means exclusionary disputes arise regarding who should be looking after patients, and resources and beds are ‘gatekept’.^2^ This superficial and transactional form of caring is perpetuated throughout medical education and training too, for example in the form of examinations that promote a performative etiquette style of empathy to tick the boxes of a mark scheme. Service structure in the UK also means that families and communities are not expected to, and are often discouraged from, participating in caring for their loved ones.^9^ It seems that the value granted to the carer decreases the closer they get to physically taking care of another, in comparison to clinicians who often play the role of determining a high-level ‘care’ plan.^10^ These factors combine to show how the biomedical approach takes precedence over psychosocial-spiritual well-being. Pharmacological therapies are over-deployed to stick a temporary plaster on distress, without considering the possibilities of caring and healing that go beyond to address the root causes of people's distress.

Familism, integral to many Global South cultures, counters the individualistic approach of Western psychiatry. Although family dynamics can contribute to intergenerational distress and trauma, the crucial role of family in offering security, solidarity, a support network and a safe space cannot be overlooked. In communities with strong familial bonds, relying on family support during distress is both anticipated and essential.

An example of a familism-orientated practice, where familial relationships and stories that pass down through generations are used as therapy, is cuento therapy, which originated in Puerto Rican and other Latin American communities.^11^ ‘Cuentos’, traditional folktales told by mothers to their children, are used as a psychotherapy device to increase awareness of and pride in the person's culture of origin, increase self-esteem and social judgement skills, and reduce anxiety. As a culturally sensitive modality of therapy, the same method of sharing narratives can be incorporated with stories from other cultures too.^11^

Similarly, within South Asian communities, people often turn initially to their families for solace. On top of the collective sense of responsibility and interdependence between families, there is often a shared spiritual or religious understanding. Clinicians in India recognise the importance of spiritual healing, working alongside the biomedical, and not against it – leading to some centres specialising in religious and spiritual healing being regarded as preferable to modern Western psychiatric treatment.^3^ The concept of collective compassionate kindness *Samastha Lokah Sukhino Bhavanthu*, Sanskrit for ‘Let all the beings in all the worlds be happy’,^12^ is central to moral psychology in Buddhism and Hinduism. Integrative cultural disciplines such as yoga and meditation exemplify a holistic ‘mind, body and soul’ approach to distress. The Bapu Trust is an example of an organisation campaigning to re-establish spiritual practices, which have become seen as ‘alternatives’ or redundant, back at the centre of caring, to prioritise local and indigenous wisdoms and maintain cultural diversity of the mind.^13^

## Conclusion

Circling back to psychiatry in the UK, transformations in mindsets need to take place. UK psychiatry needs to unlearn its canonical bias towards the reductive and hyper-individualistic approach to diagnosis. Rather, distress needs to be contextualised to acknowledge it as a response to socio-politico-economic injustices.

UK psychiatry also needs to unlearn its bias towards prescribing temporary pharmacological fixes within a fragmented system. By giving way to a practice that nurtures caring communities and collective responsibility, the value of familism and expanded forms of healing can be embraced. This will allow for authentic, integrative compassion to be at the centre of mental healthcare.

It is crucial to highlight that lessons raised from other countries and cultures are historically, politically and socially specific. UK psychiatry cannot simply copy and paste these cases into its current system, as a ‘mere indigensation’.^14^ UK psychiatry must respect and acknowledge the communities it co-creates with, to not propagate its colonial imposition and erasure of indigenous wisdoms from the Global South. By critiquing both its current systems and lessons from around the world, ideologies can be adapted to its immediate context.^14^

As a society, we must come together to realise how entrenched structural injustices in current UK culture affect us all, but also recognise that we play a role in shaping this. Across the spectrum of patients, clinicians, policymakers, communities and systems, caring needs to be informed by the pursuit of social justice. We must each recognise our actions as activism and join the movement to unlearn our biases, decolonise our minds and effect change as praxis.

## Data Availability

Data availability is not applicable to this article as no new data were created or analysed in this study.

## About the author

At the time of writing, Venkatalakshmi Satram was a 5th year medical student, now in the 6th year, in the Faculty of Medicine at Imperial College London, London, UK.
